# Serum osteocalcin as a novel biomarker for differentiating growth hormone deficiency from idiopathic short stature

**DOI:** 10.3389/fendo.2026.1745971

**Published:** 2026-03-11

**Authors:** Yuling Wang, Huanzhen Zhou, Hongye Wang, Jianying Ma, Xingyu Chen, Hongmei Yang, Hongcun Zheng, Aiping Wang

**Affiliations:** 1 Kunming First People’s Hospital, Affiliated to Kunming Medical University, Kunming, Yunnan, China; 2 Clinical Medical College of Dali University, Dali, Yunnan, China

**Keywords:** bone turnover markers, diagnostic markers, growth hormone deficiency, idiopathic short stature, osteocalcin

## Abstract

**Background:**

The differentiation between Growth Hormone Deficiency (GHD) and Idiopathic Short Stature (ISS) primarily relies on the growth hormone stimulation test (GHST),which is invasive and can cause adverse effects.

**Objective:**

To evaluate the diagnostic value of bone turnover markers in distinguishing GHD from ISS.

**Methods:**

A cross-sectional study was conducted, enrolling 76 children aged 3–11 years with short stature (37 in the GHD group and 39 in the ISS group). Clinical data including height, weight, bone age, Insulin-like Growth Factor 1(IGF-1), and Peak Growth Hormone (GHP) levels were collected. Eight bone turnover markers were measured: Osteocalcin (OC), β-C-terminal telopeptide of type I collagen (β-CTX), 25-Hydroxyvitamin D (25(OH)D), Vitamin D (VitD), Alkaline Phosphatase (ALP), Parathyroid Hormone (PTH), serum Calcium (Ca), and serum Phosphorus (P). Nonparametric tests were used for intergroup comparisons. Logistic regression and Receiver Operating Characteristic (ROC) curve analyses were performed to assess diagnostic efficacy, and Spearman correlation analysis was employed for correlation evaluation.

**Results:**

The OC level in the GHD group was significantly lower than that in the ISS group (P < 0.001), while serum P was higher in the GHD group (P < 0.05). Multivariate analysis identified OC as an independent discriminative factor (OR = 182.585, P < 0.001). ROC curve analysis revealed that OC had an area under the curve (AUC) of 0.949, At a cutoff of 1.026 ng/mL, sensitivity was 86.49% and specificity was 100%. Correlation analysis indicated a positive association between OC levels and GHP (r = 0.6, P < 0.05).

**Conclusion:**

Serum OC shows high diagnostic value for distinguishing GHD from ISS, demonstrating significant clinical utility.

## 1 Introduction

Short stature is defined as a height more than 2 standard deviations below the mean for age, sex, and ethnicity (1). It can lead to various chronic systemic diseases and psychological disorders, and may even affect the physical development of offspring, making it an important public health concern. Based on etiology, genetic background, and clinical manifestations, short stature can be classified into several types including Growth Hormone Deficiency (GHD), Idiopathic Short Stature (ISS), Small for Gestational Age (SGA), Turner syndrome, and Prader-Willi syndrome, among which GHD and ISS are the most common (2). Studies have shown that recombinant human Growth Hormone (rhGH) can significantly improve growth velocity and final height in children with GHD, particularly when diagnosed and treated early, but its efficacy is more limited in ISS patients (3, 4). Therefore, early diagnosis of GHD is crucial for optimal treatment outcomes (5).

Currently, the diagnosis of GHD is based on a comprehensive evaluation including medical history, physical measurements, growth hormone stimulation test (GHST), biochemical tests, radiological assessment of skeletal maturity, and pituitary neuroimaging, while ISS is diagnosed by excluding other known causes (6). The differentiation between GHD and ISS primarily relies on Peak Growth Hormone (GHP) levels in GHST. However, the combined stimulation test requires strict conditions and multiple invasive procedures, with potential complications such as hypoglycemia and hypotension during the test, which may reduce patient compliance. Therefore, identifying novel diagnostic markers for differentiating these conditions is of great importance for the diagnosis and treatment of short stature (7, 8). A cross-sectional study by Ye Yong et al. has demonstrated that pituitary morphology can be used to distinguish GHD from ISS (9). However, considering the high cost and practical challenges of MRI, a simpler, cost-effective diagnostic marker is needed.

Bone Turnover Markers (BTMs) are biochemical substances released during bone formation and resorption, reflecting the dynamic balance of bone metabolism (10). In recent years, the application of BTMs in childhood growth disorders has gained increasing attention. A study on BTMs during GH treatment in SGA children showed that compared with normal children, GHD and SGA children had lower bone formation markers and higher bone resorption markers (11). BTMs promote longitudinal bone growth by participating in osteoblast and osteoclast activities, a process regulated by the GH-IGF-1 axis, the core regulatory system of height (12–14). It has been well established that GHD patients have Growth Hormone (GH) deficiency (GHP <10 ng/mL in stimulation tests), while ISS patients do not (GHP >10 ng/mL) (15, 16). Therefore, we hypothesize that as downstream effectors of GH-IGF-1 axis activity, BTMs may show different concentrations in GHD and ISS children, potentially serving as discriminative markers for these two conditions.

To test this hypothesis, we measured eight BTMs, Osteocalcin (OC), β-C-terminal telopeptide of type I collagen (β-CTX), 25-Hydroxyvitamin D (25(OH)D), Vitamin D (VitD), Alkaline Phosphatase (ALP), Parathyroid Hormone (PTH), serum Calcium (Ca), and serum Phosphorus (P) in GHD and ISS children of the same age group, along with collection of clinical data including age, height, weight, bone age, Insulin-like Growth Factor 1(IGF-1), and GHP levels, aiming to identify BTMs that can differentiate GHD from ISS.

## 2 Research methods

### 2.1 Study design and participants

**Figure 1:**
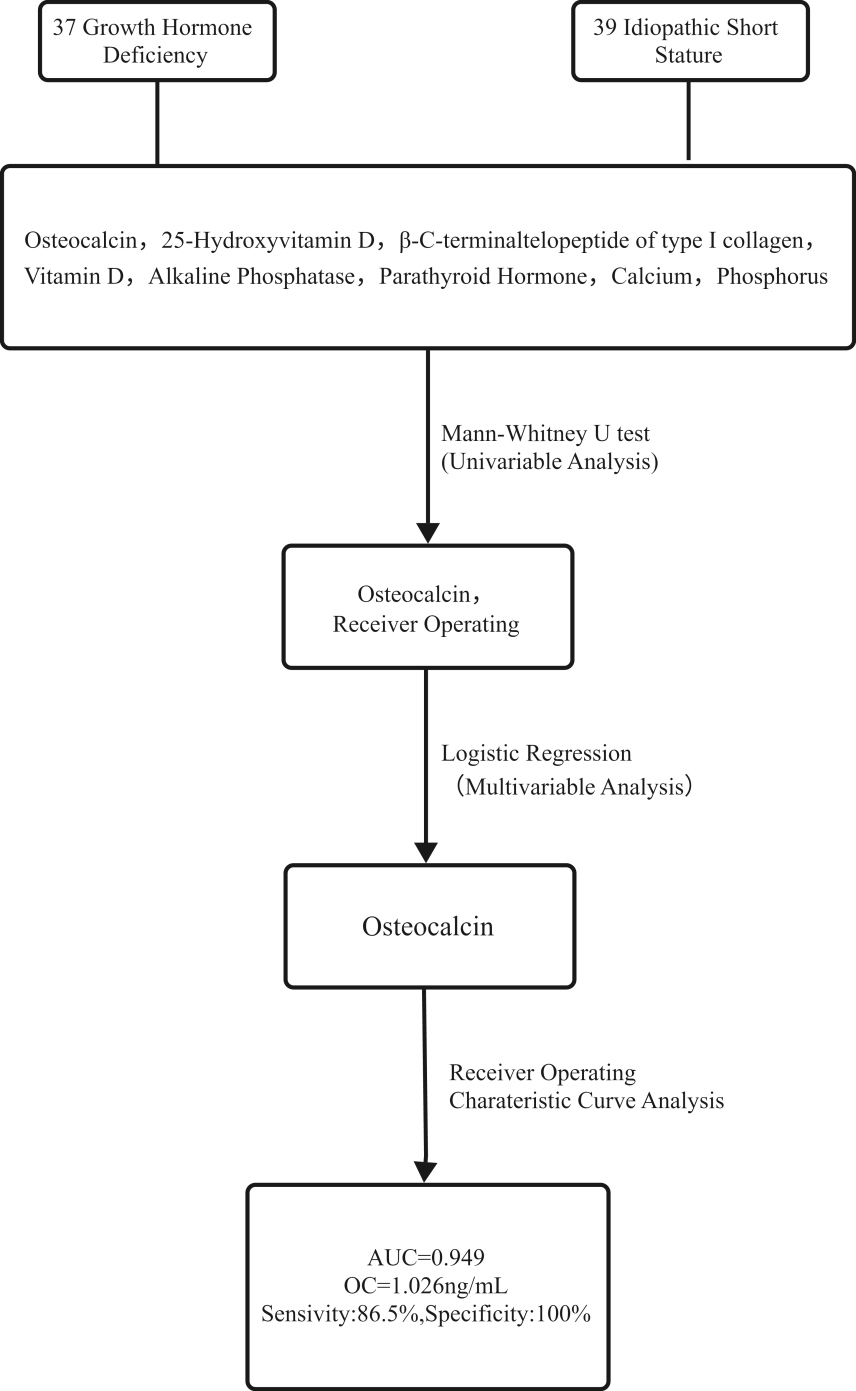
Technical road map. Notes: A panel of eight bone turnover markers was analyzed in 76 children with short stature (37 GHD, 39 ISS). Univariate analysis identified Osteocalcin (OC) and Phosphorus (P) as significantly different between groups. These were included in a multivariable logistic regression model, which confirmed OC as an independent predictor. The diagnostic performance of OC was then evaluated using ROC curve analysis.

This cross-sectional study randomly enrolled treatment-naïve patients aged 3–11 years initially diagnosed with GHD or ISS at the Pediatric Growth and Development Management Center of Kunming First People’s Hospital from October 2024 to June 2025. The study was approved by the hospital’s Ethics Committee. Additionally, as the blood samples used in this study were all residual clinical samples, the requirement for obtaining informed consent was waived. The technical roadmap is illustrated in **Figure 1**.

#### 2.1.1 Inclusion criteria: age: 3–11 years

The diagnostic criteria for the GHD group and ISS group were based on the standards outlined in “Recommendations for the clinical use of rhGH in children” (17). The specific diagnostic criteria are as follows:

Diagnostic Criteria for GHD: ①Height below the third percentile (–1.88 SDs, –1.88 SD) or –2 SDs (–2SD) for age and gender in normal healthy children. ②Annual growth velocity <7 cm/year (under 3 years of age); <5 cm/year (3 years to pre-puberty); <6 cm/year (during puberty). ③Proportional short stature with immature facial features. ④Normal intellectual development. ⑤Delayed bone age compared to chronological age. ⑥GHP levels <10 μg/L in two pharmacological GH stimulation tests; and serum IGF-1 level below the normal range.

Diagnostic Criteria for ISS: ISS was defined according to the referenced clinical recommendations (17) as a height more than 2 SDs below the mean (–2 SD) for children of the same sex and chronological age in a normal population, after the exclusion of other causes of short stature, including GHD, SGA, systemic diseases, other endocrine disorders, nutritional deficiencies, chromosomal abnormalities, skeletal dysplasias, and psychosocial or emotional disturbances.

#### 2.1.2 Exclusion criteria

(1) Children with genetic or metabolic disorders known to cause growth retardation (e.g., Turner syndrome, Prader-Willi syndrome, Noonan syndrome), chronic systemic diseases (e.g., chronic kidney disease, congenital heart disease), endocrine abnormalities (e.g., hypothyroidism, Cushing’s syndrome, diabetes mellitus), skeletal dysplasias, or psychosocial disorders (e.g., severe malnutrition, emotional deprivation syndrome) were excluded (18, 19).

(2) Children with conditions affecting bone metabolism such as rickets, metabolic bone diseases, rheumatoid arthritis, or malignant tumors were excluded. Those who had recently received medications influencing bone metabolism, including bisphosphonates or glucocorticoids, were also excluded (19).

### 2.2 Data collection

#### 2.2.1 Clinical data collection

1. Age, sex, height, weight, and BMI (kg/m²): Height and weight were measured following WHO standard procedures for assessing child growth indicators. BMI was calculated as weight (kg) divided by height squared (m²).
2. Bone age: An anteroposterior X-ray of the left hand and wrist was obtained. Bone age was assessed using the Greulich-Pyle method, and the Bone Age Index (BAI) was calculated as bone age divided by chronological age.
3. GHP and IGF-1

All participants fasted from midnight. The growth hormone stimulation test (GHST), comprising insulin-induced hypoglycemia (the insulin tolerance test, ITT) and a combined arginine plus clonidine test. The ITT was employed as a robust physiological stimulus under strict safety protocols, in line with its selective use in diagnostic guidelines for complex cases (20). All tests were conducted under continuous medical supervision.

#### 2.2.2 Bone turnover markers

After an 8-hour fast, blood samples were collected from all participants between 8:00 and 10:00 AM. OC and β-CTX were measured using electrochemiluminescence immunoassay (Roche e411, Switzerland) with original Roche reagents. The OC assay detects the intact molecule as well as the large N-MID fragment, with reported reference intervals being age and sex-specific, which explains the lower absolute values in our pediatric cohort compared to adult ranges. 25(OH)D was quantified by Liquid Chromatography–tandem Mass Spectrometry (LC-MS/MS). ALP was analyzed using the rate method, PTH and VitD were measured by chemiluminescence immunoassay, and Ca and P were determined via colorimetric assays.

### 2.3 Statistical analysis

Data were analyzed using SPSS version 26.0. Continuous variables with non-normal distribution are presented as median (interquartile range) and compared using non-parametric tests (Mann-Whitney U test for intergroup differences). Spearman’s correlation was applied for correlation analysis. Normally distributed data are expressed as mean ± SD and compared using the independent samples t-test. Pearson’s correlation was used for normally distributed variables. Logistic regression analysis was performed to identify bone turnover markers associated with GHD and ISS. Diagnostic performance was evaluated using receiver operating characteristic (ROC) curve analysis. A two-tailed P-value < 0.05 was considered statistically significant.

## 3 Results

### 3.1 Baseline clinical characteristics

A total of 76 children aged 3–11 years with short stature were enrolled according to the inclusion/exclusion criteria, comprising 37 GHD cases and 39 ISS cases. The GHD and ISS groups showed no significant differences in sex distribution, age, BMI, or BAI (all P>0.05). However, the GHD group demonstrated significantly lower IGF-1 levels and GHP values compared to the ISS group (P<0.05). Detailed baseline characteristics are presented in **Table 1**.

**Table 1:** Baseline characteristics of 76 pediatric patients.

Characteristics	GHD(N = 37)	ISS(N = 39)	*P value*
Gender			0.76
Male,n(%)	25 (32.89%)	24 (31.58%)	
Female,n(%)	12 (15.79%)	15 (19.74%	
Age(years)	6.00 (5.00-8.00)	6.00 (5.00-8.25)	0.52
Height(cm)	103.50 (96.90-113.70)	108.70 (98.20-118.10)	0.26
Weight(Kg)	15.95 (14.25-18.10)	16.90 (14.45-21.77)	0.32
BMI(Kg/m^2^)	15.10 (13.90-15.61)	14.70 (14.10-15.65)	0.68
BAI	0.65 (0.50-0.83)	0.72 (0.60-0.84)	0.24
IGF-1(ng/mL)	92.60 (58.60-119.60)	113.00 (74.35-160.40)	0.04
GHP(μg/L)	7.74 (6.41-8.95)	15.46 (12.20-19.55)	<0.001

Note: Data are presented as Median (Interquartile Range, IQR). Statistical comparisons for continuous variables were performed using Mann-Whitney U test, and categorical data (Gender) were compared using the Chi-square test. Abbreviations: BMI, body mass index; BAI, bone age index; IGF-1, insulin-like growth factor 1; GHP, growth hormone peak. Height SDS was calculated based on Chinese pediatric growth standards. An SDS of ≤ -2 corresponds approximately to the 3rd percentile.

### 3.2 Differences in BTMs between GHD and ISS groups

**Figure 2:**
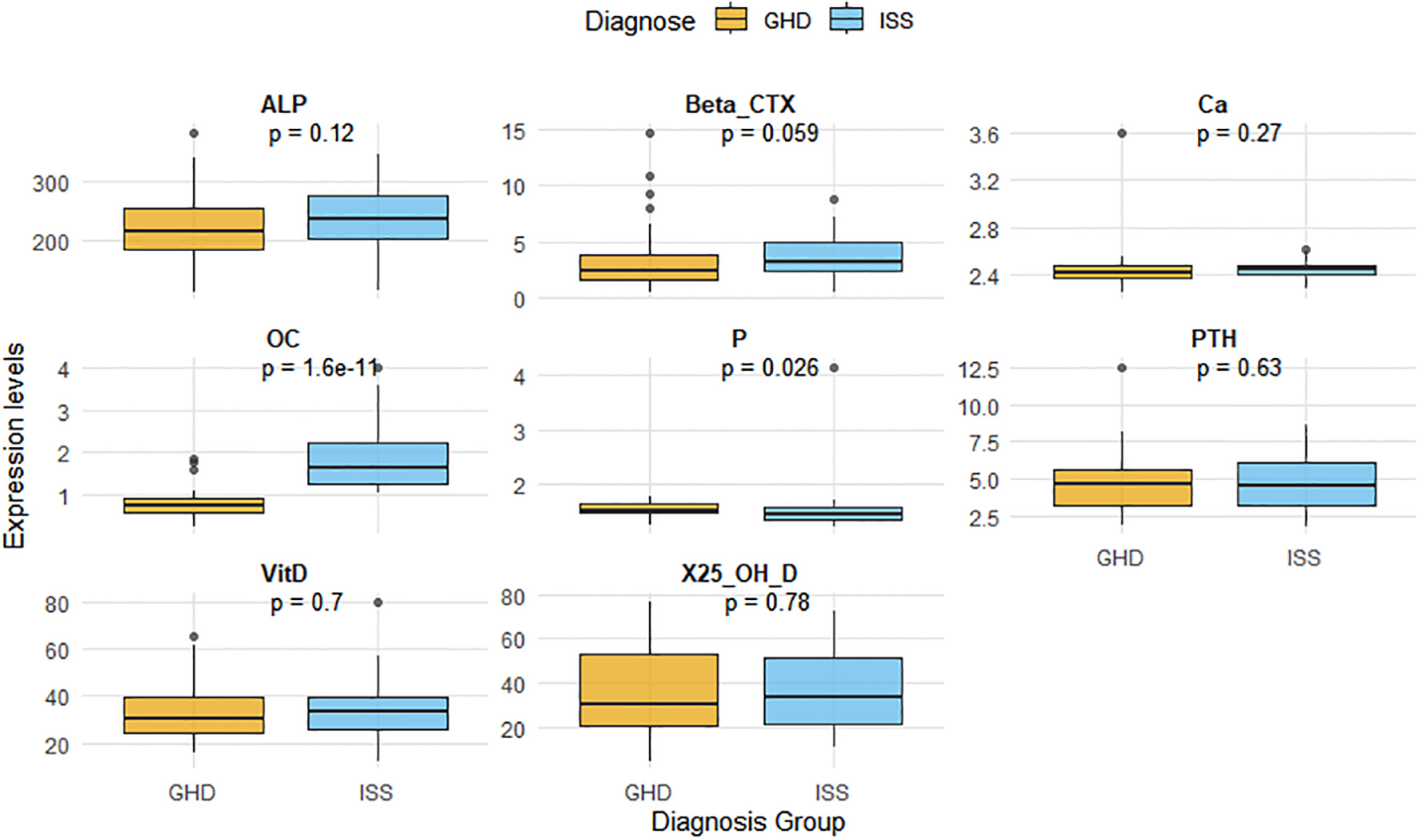
Comparison of BTMs between the GHD and ISS groups. Notes: The diagram lists the eight Bone Turnover Markers and their respective units of measurement that were analyzed. GHD, growth hormone deficiency; ISS, idiopathic short stature; OC, osteocalcin; β-CTX, β-C-terminal telopeptide of type I collagen; 25(OH)D, 25-hydroxyvitamin D; VitD, vitamin D; ALP, alkaline phosphatase; PTH, parathyroid hormone; Ca,serum calcium; P,serum phosphorus.

Eight BTMs were analyzed: OC, β-CTX, 25(OH)D, VitD, ALP, PTH, Ca, and P. The GHD group showed significantly lower OC levels (P<0.001) and higher serum P levels (P<0.05) compared to the ISS group. No significant differences were observed in β-CTX, 25(OH)D, ALP, PTH, or Ca levels between groups (all P>0.05). The results of the intergroup comparative analysis of bone turnover markers are presented in **Figure 2**, **Table 2**.

**Table 2:** Table of differential expression analysis of 8 BTMs.

Characteristics	GHD (N = 37)	ISS (N = 39)	*P- value*
OC(ng/mL)	0.744 (0.575-0.917)	1.629 (1.281-2.247)	<0.001
β-CTX(ng/mL)	2.287 (1.549-3.778)	3.161 (2.396-4.927)	0.06
25(OH)D(ng/mL)	30.501 (20.435-53.145)	33.327 (21.078-51.642)	0.78
VitD(ng/mL)	30.540 (24.480-39.890)	33.550 (25.470-39.620)	0.7
ALP(unit/L)	217.000 (185.000-254.000)	237.000 (205.000-276.000)	0.12
PTH(pmol/L)	4.670 (3.180-5.640)	4.520 (3.235-6.095)	0.63
Ca(mmol/mL)	2.420 (2.370-2.480)	2.450 (2.400-2.480)	0.27
P(mmol/mL)	1.520 (1.480-1.640)	1.460 (1.370-1.585)	0.03

Note: Data are presented as Median (Interquartile Range, IQR). Statistical comparisons for continuous variables were performed using Mann-Whitney U test. Abbreviations: OC, osteocalcin; β-CTX, β-C-terminal telopeptide of type I collagen; 25(OH)D, 25-hydroxyvitamin D; VitD, vitamin D; ALP, alkaline phosphatase; PTH, parathyroid hormone; Ca, calcium; P, phosphorus.

### 3.3 Multivariate analysis of clinical parameters and bone turnover markers

Using the diagnosis of GHD versus ISS as the dependent variable and bone metabolism markers that showed statistically significant differences in univariate analysis (OC and P) as independent variables, logistic regression analysis was performed. The analysis identified OC as a significant influencing factor for distinguishing between GHD and ISS. The results of the logistic regression analysis are presented in **Table 3**.

**Table 3:** Logistic regression analysis of BTMs for discriminating between GHD and ISS.

Variable	Coefficient	Std. error	Wald	*P value*	OR (95%CI)
OC	5.207	1.252	17.301	<0.001	182.585(15.698-2123.725)
P	0.088	1.045	0.007	0.932	1.092(0.141-8.472)

Note: This table presents the results of the multivariable logistic regression model with the diagnosis of GHD as the dependent variable. The model included Osteocalcin (OC) and Phosphorus (P) as independent variables, which were identified as significant in the prior univariable analysis. Abbreviations: OR, odds ratio; CI, confidence interval; Std. Error, standard error. Interpretation: Osteocalcin was a strong and statistically significant independent predictor of GHD (OR = 182.59, 95% CI: 15.70–2123.73; P < 0.001). In contrast, serum Phosphorus level was not an independent predictor in this model (P = 0.932).

### 3.4 Correlation between BTMs and clinical baseline parameters in children with GHD and ISS

**Figure 3:**
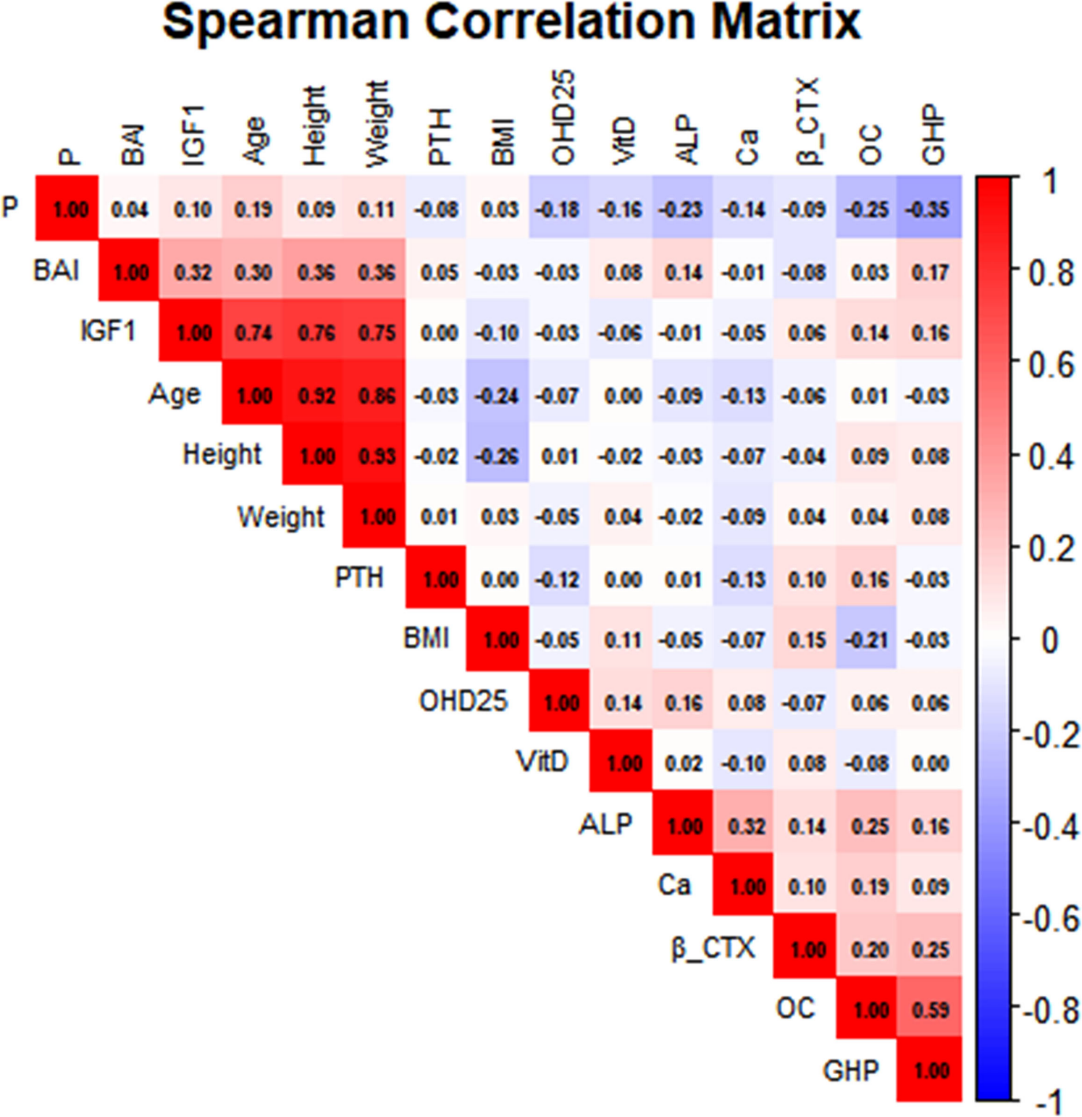
Correlations between BTMs and baseline characteristics in children with GHD and ISS. The figure presents the correlation coefficients (upper value in each cell) and the corresponding -log10(p-value) (lower value in each cell) for pairwise associations among the measured variables. A higher -log10(p-value) indicates greater statistical significance. The analyzed parameters include, GHD, growth hormone deficiency; ISS, idiopathic short stature; BMI, body mass index; BAI, bone age index; IGF-1, insulin-like growth factor-1; GHP, peak growth hormone; OC, osteocalcin; β-CTX, β-C- terminal telopeptide of type I collagen; 25(OH)D, 25-hydroxyvitamin D; VitD, vitamin D; ALP, alkaline phosphatase; PTH, parathyroid hormone; Ca, serum calcium; P, serum phosphorus.

**Figure 4:**
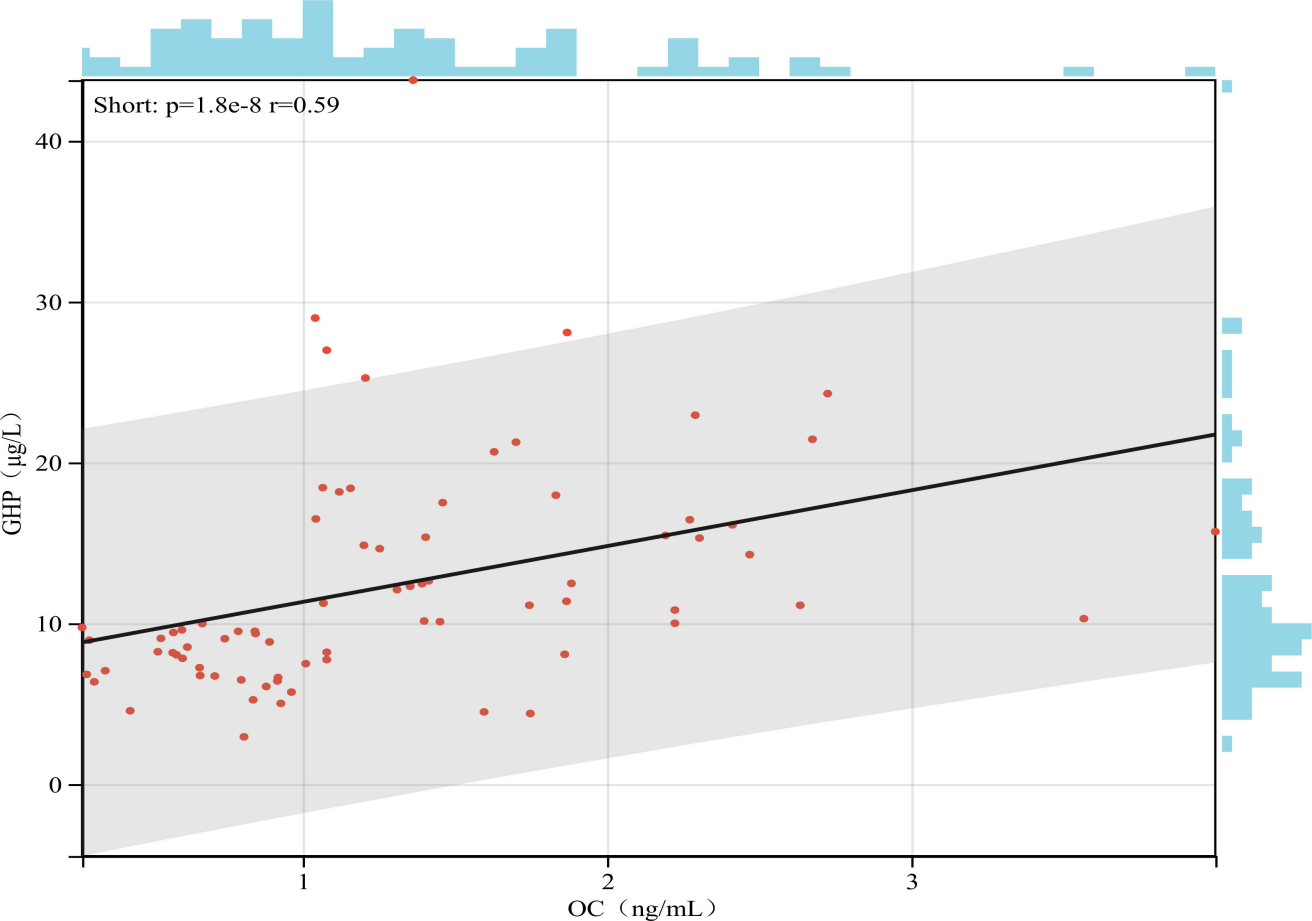
Correlation between OC and GHP levels in children with GHD and ISS. Note: Scatter plot showing the positive correlation across all participants (n=76). The solid blue line represents the linear regression fit, and the gray shaded area indicates the 95% confidence interval.

In both groups, the levels of OC and β-CTX showed a positive correlation with the GHP(r = 0.6 and r = 0.24, respectively; P < 0.05 for both). In contrast, serum P levels were negatively correlated with GHP (r = -0.35, P < 0.05). The results of the correlation analysis are presented in **Figure 3** and **4**.

### 3.5 Diagnostic performance of OC in differentiating GHD from ISS

**Figure 5:**
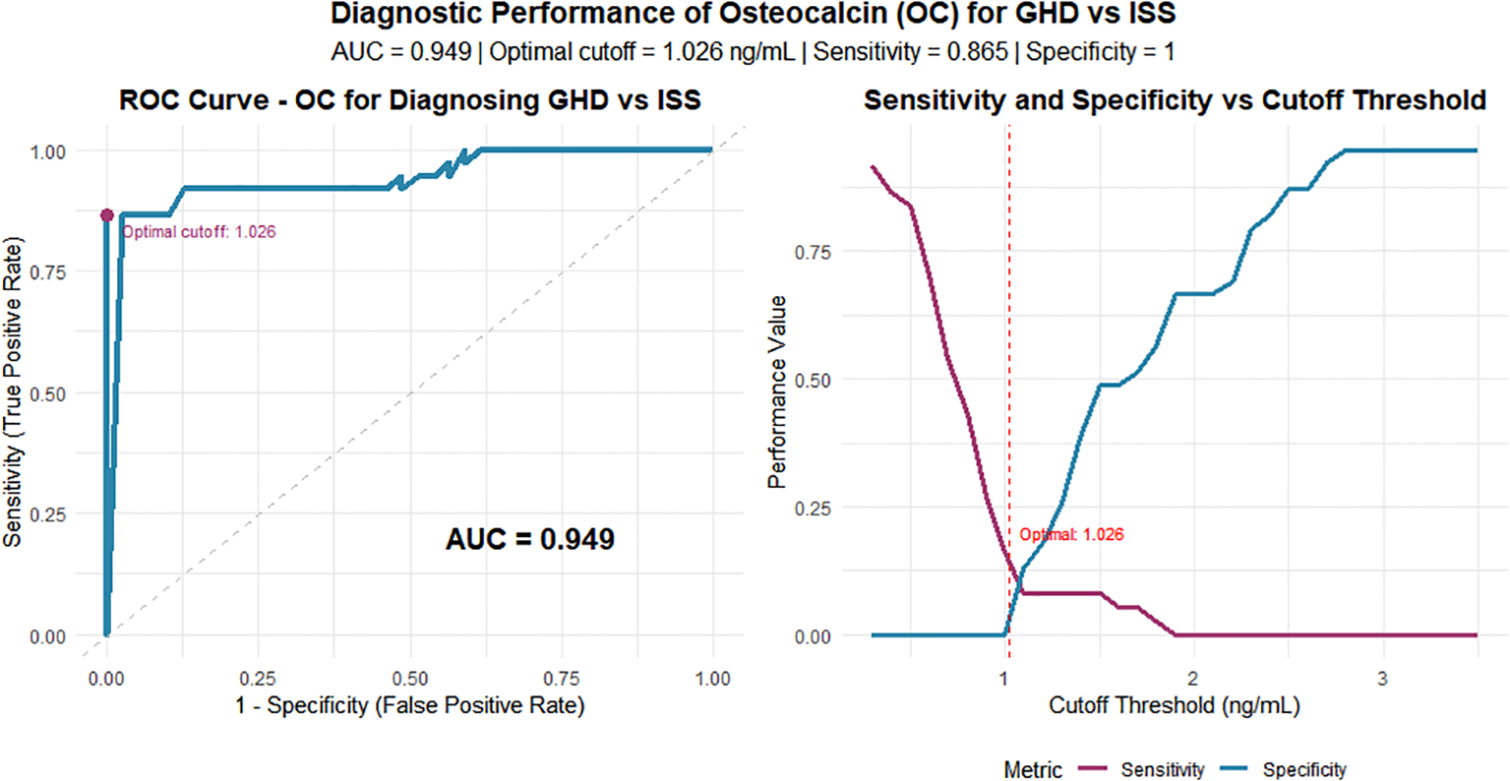
The diagnostic efficacy of OC in discriminating between ISS and growth GHD. **(A)** Receiver Operating Characteristic (ROC) curve for serum OC. The Area Under the Curve (AUC) is 0.949. The optimal cut-off point (1.026 ng/mL) is indicated. **(B)** The variation of sensitivity and specificity across different OC threshold values, highlighting the optimal cut-off.

The ROC curve analysis indicated that serum OC has high diagnostic value in distinguishing GHD from ISS, with an area under the curve (AUC) of 0.949(The differential diagnostic performance of 8 BTMs is shown in **Table 4**. Using the maximum Youden index, the optimal cutoff was 1.026 ng/mL, yielding a sensitivity of 86.5% and a specificity of 100%.See **Figure 5**.

**Table 4:** Diagnostic performance of BTMs discriminating GHD from ISS.

Marker	AUC	AUC_CI	Sensitivity	Specificity
OC (ng/mL)	0.949	(0.898-1.000)	100.00%	86.50%
P (mmol/mL)	0.649	(0.523-0.774)	75.70%	56.40%
β-CTX (ng/mL)	0.626	(0.495-0.758)	71.80%	62.20%
ALP (U/L)	0.605	(0.477-0.734)	53.80%	67.60%
Ca (mmol/mL)	0.573	(0.442-0.705)	76.90%	40.50%
PTH (pmol/L)	0.532	(0.401-0.664)	64.10%	45.90%
Vitamin D (ng/mL)	0.526	(0.394-0.658)	53.80%	59.50%
25(OH)D (ng/mL)	0.519	(0.387-0.651)	56.40%	54.10%

Note: Diagnostic performance was evaluated using receiver operating characteristic (ROC) curve analysis. The AUC represents overall diagnostic accuracy, where a value of 1.0 indicates perfect discrimination and 0.5 indicates no discriminative value. Serum OC demonstrated superior diagnostic efficacy with the highest AUC (0.949), achieving 100% sensitivity and 86.5% specificity at its optimal cut-off. The performance of other markers was comparatively limited (AUC range: 0.519–0.649). Abbreviations: AUC, area under the receiver operating characteristic curve; CI, confidence interval; OC, osteocalcin; P, phosphorus; β-CTX, β-C-terminal telopeptide of type I collagen; ALP, alkaline phosphatase; Ca, calcium; PTH, parathyroid hormone.

## 4 Discussion

Osteocalcin is known as a bone turnover marker, but its role in distinguishing GHD from ISS remains poorly studied. This study provides new evidence on the GH-IGF-1 axis in skeletal development by analyzing bone metabolism differences between GHD and ISS children, confirming GH’s critical role and identifying a potential diagnostic biomarker.

We evaluated eight BTMs. Initial analysis showed differences in OC and P. Multivariate analysis confirmed OC as an independent predictor. Its high diagnostic value (AUC 0.949) suggests strong discriminatory power. At a cutoff of 1.026 ng/mL, OC demonstrated a sensitivity of 86.49% and a specificity of 100%. While some cases might be missed, the low false-negative rate, combined with OC testing’s low cost and high reproducibility, allows for periodic monitoring. The high specificity minimizes false positives, reducing the burden of unnecessary treatment. From a practical standpoint, serum OC measurement offers advantages of widespread availability in clinical laboratories, relatively low cost compared to GHST or MRI, and simple sample collection (single venipuncture), supporting its potential feasibility as an adjunctive diagnostic tool.

Mechanistically, OC is essential for bone mineralization. Lower OC in GHD may result from impaired osteoblast function due to reduced GH activity, whereas relatively normal GH in ISS preserves OC synthesis (21). Our finding of a positive OC-GH correlation supports a GH-mediated regulation mechanism. Although P was significant in univariate analysis, it was not retained in the multivariate model, possibly because its role is encompassed within OC’s functions.

This study elucidates distinct bone metabolic profiles between GHD and ISS. The positive OC-GHP correlation aligns with known mechanisms of GH stimulating osteoblasts. Consistent with this, rhGH therapy in GHD increases OC levels alongside bone density improvements (22, 23). While GH is thought to regulate OC via IGF-1 (24), we found no OC-IGF-1 correlation, suggesting the mechanism in these children may not be primarily IGF-1-dependent. We also observed significant GH correlations with P and β-CTX, validating GH’s role in bone metabolism regulation. Interestingly, no significant correlations were found among different BTMs within each group, unlike in healthy children (25–28). This suggests disrupted bone metabolic patterns in GHD and ISS, which may contribute to short stature. However, the lack of a healthy control group limits this interpretation.

This study has limitations. First, its single-center, cross-sectional design precludes causal inferences. The proposed cut-off value of 1.026 ng/mL requires validation in larger, multicenter cohorts to confirm its robustness. Furthermore, the lack of longitudinal data limits understanding of temporal changes. Also, OC values are assay-dependent; our Roche assay results require validation with other methods. Second, unmeasured confounders like diet, sunlight, and activity could influence results. Pubertal status, though largely prepubertal, needs consideration in future studies stratified by Tanner stage. Therefore, the diagnostic cut-off should be applied alongside comprehensive clinical evaluation. Third, the age range (3–11 years) includes potential early puberty, not fully accounted for. Most importantly, this was an exploratory study without a prior sample size or power calculation. The sample size was constrained by the single-center design and the enrollment period. Therefore, while the diagnostic metrics for OC are highly promising, they must be interpreted with caution. The findings, particularly the precise cut-off value, require validation in larger, appropriately powered, prospective studies before they can be recommended for widespread clinical use. Future studies should involve larger multicenter cohorts for validation, include healthy controls, collect more covariates, and explore BTM changes during therapy.

## 5 Conclusion

This exploratory study demonstrates that serum OC has high diagnostic value for distinguishing GHD from ISS, potentially optimizing diagnostic approaches. It reveals different bone metabolic characteristics between these groups and validates the association between key BTMs and GH, enhancing our understanding of GH’s role in bone metabolism and offering a new strategy for precise diagnosis of childhood growth disorders. These promising results require confirmation in larger, prospective, and multicenter studies.

## Data Availability

The original contributions presented in the study are included in the article/supplementary material. Further inquiries can be directed to the corresponding author.
